# Effect of sugar transporter on galactose utilization in *Streptococcus thermophilus*

**DOI:** 10.3389/fmicb.2023.1267237

**Published:** 2023-11-21

**Authors:** Jiancun Zhao, Yan Liang, Susu Zhang, Zhenshang Xu

**Affiliations:** ^1^Department of Pharmacy and Biotechnology, Zibo Vocational Institute, Zibo, China; ^2^State Key Laboratory of Biobased Material and Green Papermaking, Qilu University of Technology, Shandong Academy of Science, Jinan, China; ^3^School of Bioengineering, Qilu University of Technology, Shandong Academy of Science, Jinan, China; ^4^College of Life Science, Shandong Normal University, Jinan, China

**Keywords:** *Streptococcus thermophilus*, sugar transporter, galactose utilization, lactose permease, lactose utilization

## Abstract

**Introduction:**

*Streptococcus thermophilus* is a traditional starter for dairy products. The lactose rich in milk is the main carbon source for the growth of *S. thermophilus*. However, the utilization of galactose by *S. thermophilus* is strain-specific, and many genetic factors can affect the sugar utilization phenotype of *S. thermophilus* strains.

**Methods:**

In this study, *S. thermophilus* A25, which is capable of utilizing lactose and galactose, was used as the starting strain to construct lactose permease-deficient mutant *S. thermophilus* Δ*lacS*. Subsequently, the complement vectors expressing complete lactose permease of *S. thermophilus* and its N-terminal 1–486 amino acid residues were constructed and transformed into *S. thermophilus* Δ*lacS*, respectively. Meanwhile, complement vectors expressing lactose permease and galactose/proton symporter of Escherichia coli were also constructed.

**Results and Discussion:**

Results showed that *S. thermophilus* Δ*lacS* lost the ability to utilize lactose and galactose. By measuring the growth of the recombinant strains, it was found that the strain expressing complete lactose permease of *S. thermophilus* recovered the growth ability in lactose and galactose medium, while the strain expressing N-terminal of lactose permease recovered the growth ability only in lactose medium. Furthermore, the transformation of *S. thermophilus* Δ*lacS* was not successful with the complement vector expressing *E. coli* lactose permease, while the strain expressing *E. coli* galactose/proton symporter could recover its growth ability in the galactose medium. These results suggest that the properties of sugar transporters play an important role in galactose utilization by *S. thermophilus*.

## 1 Introduction

*Streptococcus thermophilus* and *Lactobacillus bulgaricus* are the main species in the production of yogurt. These two species can quickly convert the lactose in milk into lactic acid during the yogurt fermentation process, so that the pH of milk can be rapidly reduced to promote curd, thus giving yogurt excellent texture and taste. Furthermore, *S. thermophilus* produces high-value-added secondary metabolites such as exopolysaccharides and hyaluronic acid, which give yogurt high viscosity and special nutritional value (Courtin and Rul, 2004). Although it has a close evolutionary relationship with *S. pneumoniae*, *S. pyogenes*, *S. mutans*, and other pathogenic *Streptococcus*, *S. thermophilus* has evolved into a species highly suitable for the dairy environments due to its long-term growth in dairy environment (Tettelin, 2004). The genes related to pathogenicity were deleted or pseudogenized, including adhesion genes, toxicity genes, and antibiotic modification genes (Ferretti et al., 2001; Bolotin et al., 2004). Therefore, *S. thermophilus* has the Generally Recognized as Safe (GRAS) status. To better adapt to the single carbon source in dairy products, a variety of genes related to carbon transport, degradation, and metabolism in *S. thermophilus* (about 10% of the total genes) were degraded into pseudogenes. Moreover, functional genes related to amino acid synthesis and oligopeptide transport were obtained by horizontal gene transfer to cope with the shortage of amino acids and oligopeptides in dairy products (Fernandez-Espla et al., 2000; Prajapati et al., 2013). Therefore, *S. thermophilus* has a narrow range of available carbon sources, mainly lactose, sucrose, and glucose, while the utilization of galactose and fructose is strain-specific (Hols et al., 2005).

Lactose is a disaccharide composed of α/β-D-glucose and β-D-galactose, and is the main carbon source in milk (Iskandar et al., 2019). Through the study of different strains of *S. thermophilus* and their genome sequences, the transport and metabolism of lactose and the regulatory mechanism have been deeply understood (Arai et al., 2020). The *lac* operon containing the coding gene of lactose permease LacS and β-galactosidase LacZ controls the transport and hydrolysis of lactose. *S. thermophilus* first transports lactose into the cytoplasm via LacS, which is then hydrolyzed into glucose and galactose by intracellular LacZ (Sabrina et al., 2018). Glucose is phosphorylated by glucokinase to form glucose 6-phosphate, which is then converted to pyruvate by the Embden-Meyerhof-Parnas (EMP) pathway, and pyruvate is converted to lactic acid mainly by lactate dehydrogenase. The Leloir pathway is a common galactose metabolic pathway in lactic acid bacteria. Although *S. thermophilus* has a complete Leloir pathway operon, most strains are unable to utilize galactose. The produced galactose is reversely released into the medium by permease (Xiong et al., 2019). Through the in-depth study of *S. thermophilus* strains using galactose, it was found that many factors were different from those that did not use galactose, such as the promoter sequence (Anbukkarasi et al., 2014) and ribosome binding site (Vaillancourt et al., 2004) of gal operon. The transcription and translation levels of enzymes associated with galactose metabolism eventually affected the sugar utilization phenotype of the strain. However, the galactose transport ability of *S. thermophilus* strains and the underlying genetic basis have not been explored.

*S. thermophilus* has a unique lactose uptake system, namely lactose permease, whose transport mechanism is mainly the secretion of a molecule of galactose while the cell ingests one molecule of lactose. Lactose transport can also be driven by the lactose/proton cotransport reaction if sufficient proton potential is present. Lactose permease is a member of the galactosid-pentosid-hexuronide transporter family (GPH family), which in turn belongs to the largest family of secondary transporter major facilitator superfamily (MFS). Members of the GPH family have prokaryotic or eukaryotic origins and catalyze the transport of sugars and sugar derivatives in symport with cations (Poolman et al., 1996; Reinders and Ward, 2001). These transporters consist of 12 α-helical transmembrane segments that span the membrane in a zigzag pattern. The oligomerization of LacS from *S. thermophilus* has been analyzed in detail, and this system forms a structural and functional dimer (Geertsma et al., 2005a,b). Within a LacS subunit, two domains can be distinguished. The N-terminal membrane-embedded carrier domain is responsible for catalyzing the translocation. The C-terminal hydrophilic LacS-IIA domain is homologous to the IIA^Glc^ domain of the PEP phosphotransferase system and exists on the cytoplasmic surface of the membrane. The LacS-IIA domain is not essential for transport, but plays a regulatory role. HPr (His~P) phosphorylation of LacS-IIA enables this domain to interact with the carrier domain and regulate transport activity (Poolman et al., 1995; Gunnewijk and Poolman, 2000).

The transport of lactose in *S. thermophilus* by lactose permease has been thoroughly studied, but there is no report on galactose transport in *S. thermophilus*. In this study, we constructed a lactose permease coding gene knockout strain of *S. thermophilus.* Then, the complete lactose permease of *S. thermophilus* and its N-terminal 1–486 amino acid residues were expressed to explore the effects of different domains on the transport of galactose by *S. thermophilus*. In addition, we also tried to replenish lactose permease and galactose/proton symporter derived from *E. coli* in knockout strains of *S. thermophilus*, and observed the effects of different sugar transporters.

## 2 Materials and methods

### 2.1 Strains, plasmids, culture conditions, and chemical reagents

The bacterial strains and plasmids used in this study were listed in Table 1. *E. coli* was cultured in Laura Bertani broth (1% w/v peptone, 0.5% w/v yeast extract, and 1% w/v NaCl) at 37°C with 200 rpm on a rotating oscillator. *S. thermophilus* A25 was cultured anaerobically in M17 broth (Oxoid, Basingstoke, UK) supplemented with 0.5% (w/v) lactose (LM17), galactose (M17-Galactose) or sucrose (SM17). If necessary, antibiotics were added to the medium of *E. coli* (erythromycin of 250 μg/mL or chloramphenicol of 10 μg/mL) and *S. thermophilus* (erythromycin of 2.5 μg/mL or chloramphenicol of 5 μg/mL). Bacterial genomic DNA extraction kit, Phanta max Ultra fidelity DNA polymerase, DNA purification kit, and CloneExpress II one-step cloning kit were purchased from Vazyme Biotechnology Co., LTD. (Nanjing, China). Restriction enzymes and T_4_ DNA ligases were purchased from TaKaRa Biotechnology Co., LTD. (Tokyo, Japan). Plasmid extraction kit purchased from Tiangen Co., LTD. (Beijing, China). The primers used in this study were shown in Table 2. All primers are synthesized by Sangon Biotechnology Co., LTD. (Shanghai, China).

**Table 1:** Strains and plasmids used in this study.

Strains or plasmids	Characteristics	Sources
*S. thermophilus* A25	Wild-type	Xu et al. (2022a,b)
*E. coli* XL-Blue1	Cloning host	Sangon
*E. coli* DH5α	Cloning host	Sangon
*E. coli* XL-Blue1/ pG^+^host9-*lacS*-*up-down*	*E. coli* XL-Blue1 containing pG^+^host9-*lacS*-*up-down*	This study
*S. thermophilus* A25/ pG^+^host9-*up-down*	*S. thermophilus* A25 containing pG^+^host9-*lacS*-*up-down*	This study
*S. thermophilus* Δ*lacS*	*S. thermophilus* A25 with the lactose permease gene knockout	This study
*E. coli* DH5α/ pNZ8148-*lacS*	*E. coli* DH5α containing pNZ8148-*lacS*	This study
*E. coli* DH5α/ pNZ8148-N-*lacS*	*E. coli* DH5α containing pNZ8148-N-*lacS*	This study
*E. coli* DH5α/ pNZ8148-*lacY*	*E. coli* DH5α containing pNZ8148-*lacY*	This study
*E. coli* DH5α/ pNZ8148-galR	*E. coli* DH5α containing pNZ8148-galR	This study
*S. thermophilus* Δ*lacS*/pNZ8148-*lacS*	*S. thermophilus* Δ*lacS* containing pNZ8148-*lacS*	This study
*S. thermophilus* Δ*lacS*/pNZ8148-N-*lacS*	*S. thermophilus* Δ*lacS* containing pNZ8148-N-*lacS*	This study
*S. thermophilus* Δ*lacS*/pNZ8148-*galR*	*S. thermophilus* Δ*lacS* containing pNZ8148-*galR*	This study
pG^+^host9	Temperature-sensitive plasmid, used for gene knockout in *S. thermophilus*	Maguin et al. (1992)
pG^+^host9-*lacS*-*up-down*	pG^+^host9 ligated with the upstream and homologous arm of the *lacS*	This study
pMG36e	Expression vector, containing P32 promoter	van de Guchte et al. (1989)
pNZ8148	Expression vector	Mierau and Kleerebezem (2005)
pNZ8148-*lacS*	Expression vector of complete lactose permease of *S. thermophilus*, under the P32 promoter	This study
pNZ8148-N-*lacS*	Expression vector of N-terminal lactose permease of *S. thermophilus*, under the P32 promoter	This study
pNZ8148-*lacY*	Expression vector of lactose permease of *E. coli*, under the P32 promoter	This study
pNZ8148-*galR*	Expression vector of galactose/proton symporter of *E. coli*, under the P32 promoter	This study

**Table 2:** Oligonucleotides used in this work.

Primers	Sequence(5`-3`)
*up*-F	GGTACCGGGCCCCCCCTCGAGTTCCAACGGAAACTGGTGCT
*up*-R	TTCGGAAACCTCCTATTATTTG
*down*-F	CAAATAATAGGAGGTTTCCGAATCTATGAACATGACTGAAAAAAT
*down*-R	AGTGGATCCCCCGGGCTGCAGAAGACAATTCTCTTACCATTC
*test*-F	TCGTGACTATGTGCATCC
*test*-R	GATATCAGCTGGTTTCGC
pNZ8148-F	GCTCAAGCTTTCTTTGAACC
pNZ8148-R	CCCGAGGACCGAATTCGAATTATGCTCGCGTTATCGAC
*P32*-F	TCGAATTCGGTCCTCGGG
*P32*-R	TTCAAAATTCCTCCGAATA
*lacS*-F	TTCGGAGGAATTTTGAAATGGGAAAATCTAACGGTC
*lacS*-R	CCTTCGTTTTCAGACTTTGCTTATTCTCCTTTTTTGAAGG
*N-lacS*-R	CCTTCGTTTTCAGACTTTGCTTAGTTTACAAGAGATACGACATT
*lacY*-F	TTCGGAGGAATTTTGAAATGTACTATTTAAAAAACACAAAC
*lacY*-R	CCTTCGTTTTCAGACTTTGCTTAAGCGACTTCATTCACCT
*galR*-F	TTCGGAGGAATTTTGAAATGCCTGACGCTAAAAAAC
*galR*-R	CCTTCGTTTTCAGACTTTGCTTAATCGTGAGCGCCTAT

### 2.2 Construction of gene knockout vector for *Streptococcus thermophilus*

The lab-preserved strain *S. thermophilus* A25 was inoculated into LM17 liquid medium and cultured at 42°C for 12 h. Bacterial genomic DNA extraction kit was used to extract the genome of *S. thermophilus* A25. Using the genomic DNA as a template, primer sets *up*-F/*up*-R and *down*-F/*down*-R were used to amplify the upstream and homologous arm of the target sequence, respectively. After the PCR procedure, the PCR products were detected by DNA gel electrophoresis, and the target DNA fragments were recovered. These two fragments were fused by overlapping extended PCR, and then connected to temperature-sensitive plasmid pG^+^host9 which was pretreated by restriction enzymes *Pst* I and *Xho* I. The ligated product was converted to *E. coli* XL-Blue1 and coated on LB plate containing 250 μg/mL erythromycin and then cultured at 30°C for 24 h. The transformants were selected and verified.

### 2.3 Gene knockout and validation

The knockout vector pG^+^host9-*up-down* was electroporated into *S. thermophilus* A25 according to the method of Xu et al. (2021). The strains were coated on SM17 plate containing 2.5 μg/mL erythromycin, and cultured at 30°C for 48–72 h. The transformant was selected into SM17 broth containing 2.5 μg/mL erythromycin. The plasmid was extracted and verified by PCR. For the gene first exchange, the transformants were cultured at 30°C to OD_600_ value of 0.8, and then cultured at 42°C for 2 h. Subsequently, SM17 plate containing 2.5 μg/mL erythromycin was coated and incubated overnight at 42°C. The colonies were selected and cultured at 42°C in SM17 broth containing 2.5 μg/mL erythromycin, and then the genomic DNA of the bacteria was extracted for verification. The strains with gene first exchange were continuously cultured at 30°C to occur the second homologous recombination. The above bacterial solution was diluted in a gradient, coated on a plate without antibiotics, and cultured at 42°C for 24–36 h. The colonies were selected and planted on SM17 plates with and without erythromycin. Bacterial colonies that grew on the erythromycin-free plate, but did not grow on the erythromycin plate were selected. Their genomic DNA was extracted, and primers *test*-F and *test*-R were used for PCR verification of genomic DNA of the knocked-out strain. Transformants with the target fragment size of 3,000 bp were selected.

### 2.4 Construction of complement vectors expressing different sugar transporters

The primers *lacS*-F and *lacS*-R were used to amplify the lactose permease fragment from *S. thermophilus* A25. Primers *lacS*-F and *N-lacS*-R were used to obtain the coding gene of the N-terminal 1–486 amino acid residues of lactose permease. Using the genomic DNA of *E. coli* BL21 as a template, the lactose permease of *E. coli* was amplified using primers *lacY*-F and *lacY*-R. Primers *galR*-F and *galR*-R were used to amplify the galactose/proton symporter coding gene of *E. coli*. Meanwhile, the P32 promoter fragment was amplified from pMG36e using primers *P32*-F and *P32*-R. Subsequently, the P32 promoter was fused with the above gene fragment of sugar transporters by overlapping extended PCR, and the fusion fragment was inserted into the expression vector pNZ8148. The ligated product was transformed into *E. coli* DH5α and coated on a LB plate supplemented with 10 μg/mL chloramphenicol and cultured at 37°C for 12 h. The transformants were selected and the plasmids were extracted for PCR verification. Finally, recombinant plasmids pNZ8148-*lacS*, pNZ8148-*N-lacS*, pNZ8148-*lacY* and pNZ8148-*galR* were obtained. These plasmids were electrically transferred into *S. thermophilus* Δ*lacS* to obtain recombinant strains *S. thermophilus* Δ*lacS*/pNZ8148-*lacS*, *S. thermophilus* Δ*lacS*/pNZ8148-*N-lacS* and *S. thermophilus* Δ*lacS*/pNZ8148-*galR*, respectively. The recombinant strains were screened by using SM17 plate containing 5 μg/mL chloramphenicol.

### 2.5 Electrotransformation of *Streptococcus thermophilus*

Electrotransformation of *S. thermophilus* was conducted according to the method of Xu et al. (2021). The *S. thermophilus* strain was transferred to 5 mL of LM17 and incubated at 42°C until the OD_600_ value was 0.5. An equal volume of SGSM17 (M17 broth with 0.5% sucrose, 20% glycine, and 0.8 M sorbitol) was added and incubated at 42°C for another 1 h, then ice bath for 30 min. The cultures were centrifugated at 4°C with 3,000 g for 10 min to collect bacterial cells. The cells were washed twice by using ice-cold SG buffer (0.1 M sorbitol, 10% glycerol), and resuspended in 50 μL SG buffer to obtain the competent cells of *S. thermophilus*. For electroporation, the prepared competent cells and 0.5 μg of plasmid DNA were electroporated in a 0.2-cm ice-cold cuvette at 12.50 kV per cm by using an electroporator (Eppendorf 2,510). Then, the recovery medium (M17 broth containing 0.25% sucrose, 0.4 M sorbitol, 0.02 M MgCl_2_·6H2O, and 2 mM CaCl_2_) was added and incubated at 30°C for 2 h, and then coated on the SM17 plate with corresponding antibiotics.

### 2.6 Growth determination of *Streptococcus thermophilus* strains

The recombinant strains of *S. thermophilus* were cultured overnight in SM17 supplemented with 5 μg/mL chloramphenicol. Then, these strains were inoculated (2%, v/v) into LM17 and M17-galactose, respectively, and incubated at 42°C for 24 h. Samples were taken every 2 h to determine OD_600_ to characterize the growth status of the strain, and the pH of the culture was measured with a pH meter to detect the pH change. Each experiment was done in triplicate. The mean value was calculated and the error bar represented the standard deviation of three repeated experiments.

## 3 Results

### 3.1 Construction of lactose permease-deficient mutant of *Streptococcus thermophilus*

**Figure 1:**
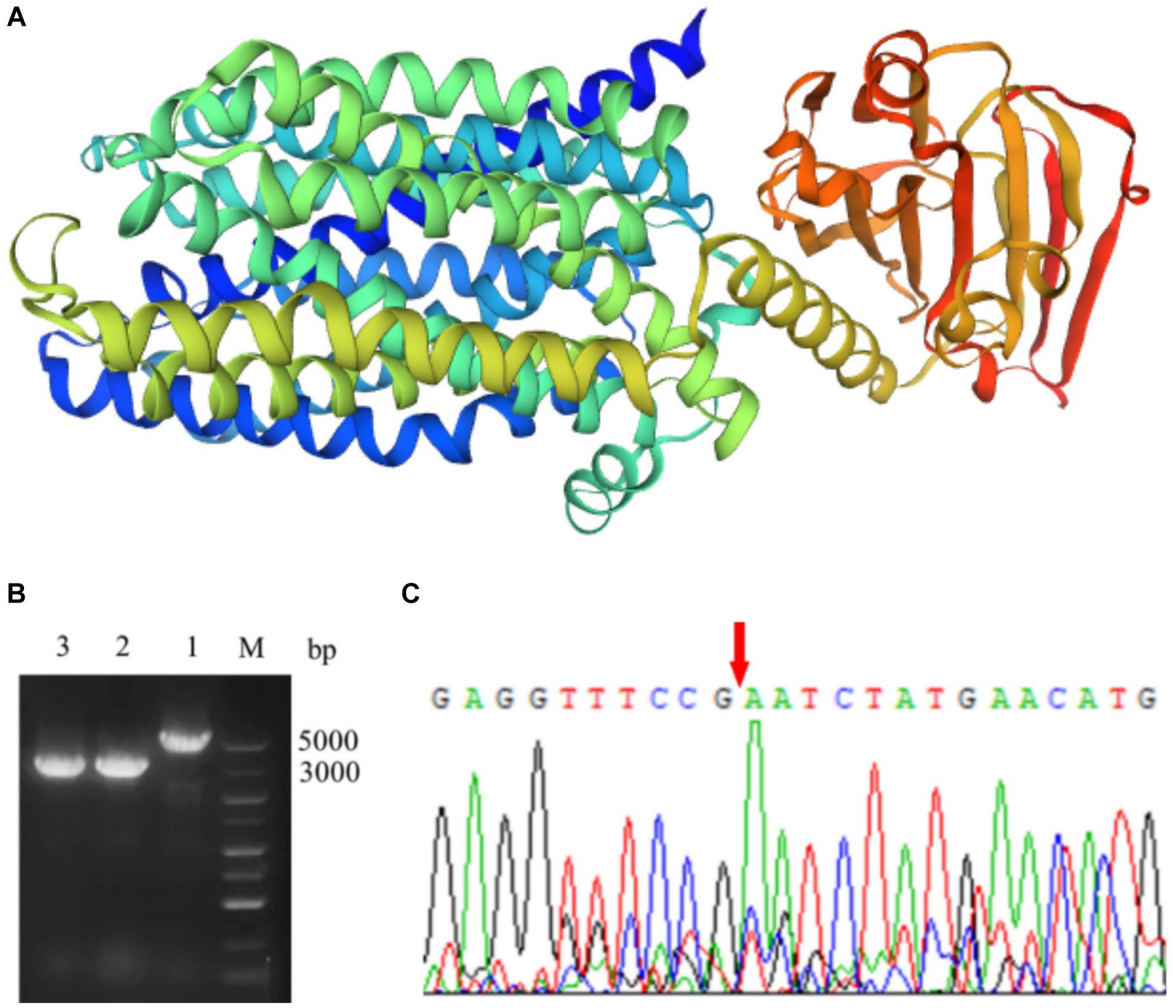
Knockout of lactose permease in *S. thermophilus*. **(A)** The protein structure simulation of LacS by AlphaFold. **(B)** PCR verification of the *lacS* deletion in *S. thermophilus*. Lane M, DL5000 DNA Marker; Lane 1, PCR product using *S. thermophilus* A25 genome as template; Lane 2 and 3, PCR product using *S. thermophilus* Δ*lacS* genome as template. **(C)** The sequencing of the target region in *S. thermophilus* Δ*lacS* genome.

The lactose permease gene of *S. thermophilus* was 1905 bp, encoding a protein of 634 amino acids (AA). As shown in Figure 1A, two independent domains which located at the N-terminal and C-terminal, respectively, were identified in LacS by using AlphaFold. The N-terminal domain belonged to the GPH sugar transporter family, and the C-terminal domain belonged to the PTS_IIA_glc superfamily. The gene deletion mutant of *S. thermophilus* A25 was constructed by using temperature-sensitive plasmid pG^+^host9. Upstream and downstream homologous arms of the *lacS* gene were amplified, fused, and ligated into the plasmid. The recombinant plasmid was introduced into *S. thermophilus* A25 by electroporation and gene knockout was performed by the natural recombination mechanism of the strain. The lactose permease-deficient mutant was named *S. thermophilus* Δ*lacS*. Genomic DNA of the mutant strain was extracted and amplified by PCR using primers *test*-F and *test*-R to confirm the gene knockout. As shown in Figure 1B, PCR product lengths of the mutant and wild strains were 3,000 bp and 5,000 bp, respectively. In addition, the sequencing results directly showed the absence of the 1905 bp target sequence (Figure 1C).

### 3.2 Differences in sugar utilization between *Streptococcus thermophilus* A25 and *Streptococcus thermophilus* ΔlacS

**Figure 2:**
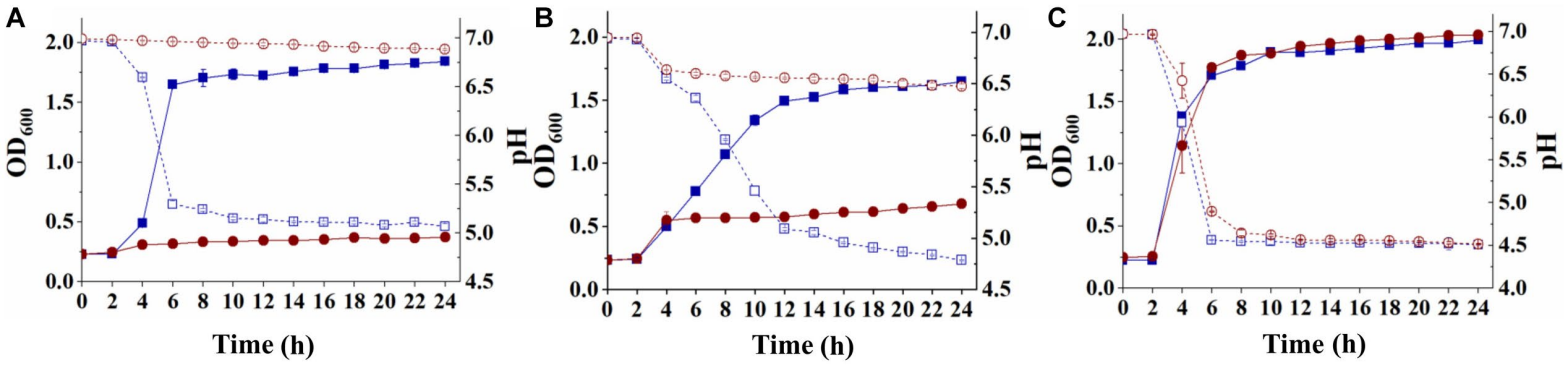
Differences in the utilization of lactose, galactose, and sucrose by *S. thermophilus* A25 (square) and *S. thermophilus* Δ*lacS* (circle). **(A)** The OD_600_ (solid line) and pH (dot line) of strains grown in LM17 medium. **(B)** The OD_600_ (solid line) and pH (dot line) of strains grown in M17-galactose medium. **(C)** The OD_600_ (solid line) and pH (dot line) of strains grown in SM17 medium.

*Streptococcus thermophilus* A25 and *S. thermophilus* Δ*lacS* were inoculated into LM17, SM17, and M17-galactose for 24 h, respectively, to analyze their growth ability under different carbon sources. As shown in Figure 2A, when cultured in a medium containing lactose as a carbon source, the OD_600_ value of the wild strain was 1.84 ± 0.02, and the pH decreased from 6.96 ± 0.01 to 5.07 ± 0.04. The OD_600_ value of the knockout strain was only 0.37 ± 0.01, and the pH was 6.89 ± 0.01. When the strains were grown in a medium containing galactose as a carbon source, their growth status was similar to that of lactose. The OD_600_ and pH of the wild strain were 1.65 ± 0.01 and 4.79 ± 0.01, respectively. The knockout strain hardly grew in galactose, with OD_600_ eventually reaching only 0.68 ± 0.03 and a final pH of 6.48 ± 0.02 (Figure 2B). However, these two strains obtained similar levels of biomass in SM17 medium, with OD_600_ values of 1.99 ± 0.01 and 2.03 ± 0.01, respectively, and pH values of 4.51 ± 0.01 and 4.52 ± 0.01, respectively (Figure 2C). These results suggested that the lactose permease-deficient mutant *S. thermophilus* Δ*lacS* lost the ability to grow in lactose and galactose medium, but the strain could still utilize sucrose, which was beneficial for the screening of recombinant strains.

### 3.3 Construction and transformation of complement vectors expressing different sugar transporters

**Figure 3:**
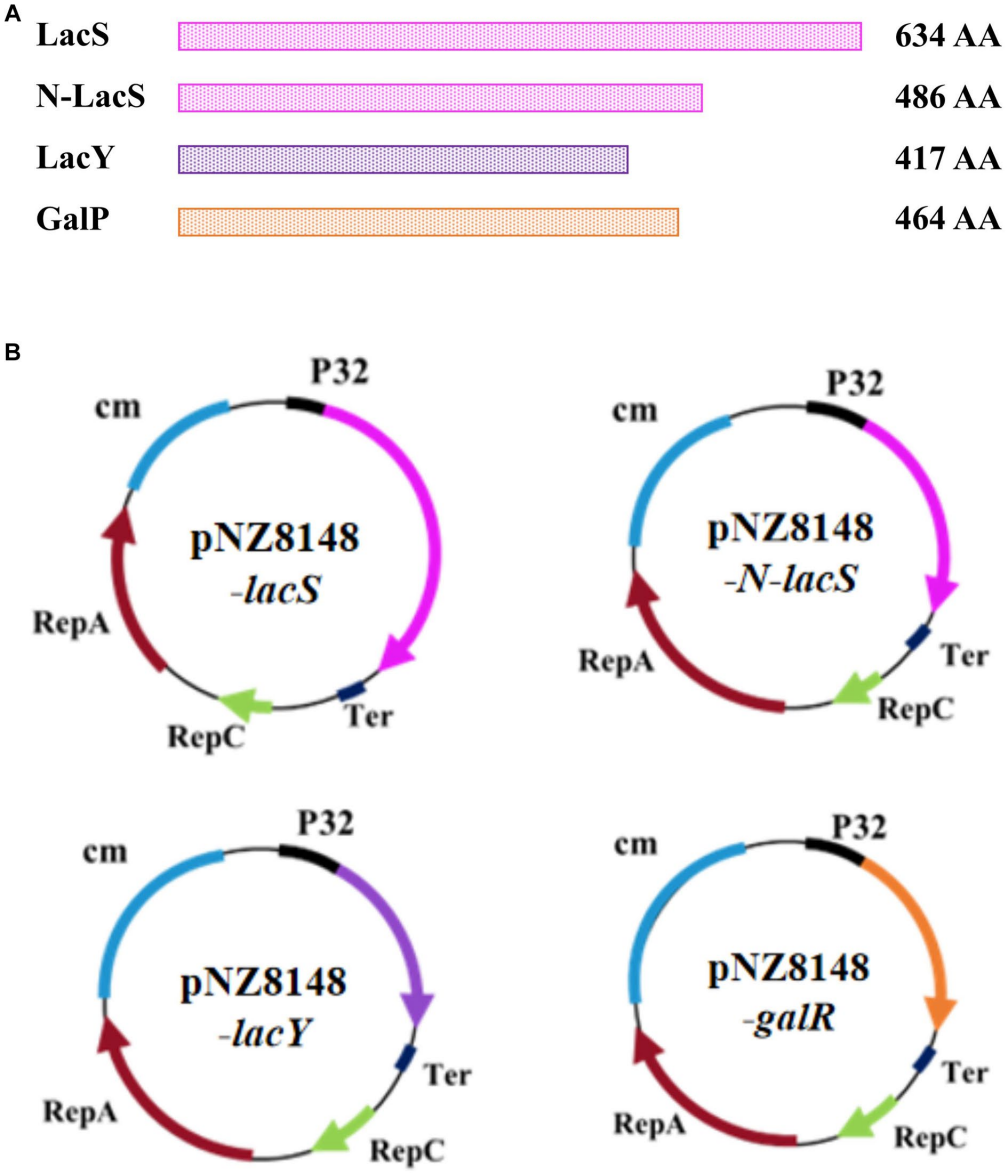
Construction of complementation vectors for *S. thermophilus* Δ*lacS*. **(A)** The schematic diagram of sugar transporters, including the lactose permease of *S. thermophilus*, the N-terminal domain of the lactose permease of *S. thermophilus*, the lactose permease of *E. coli*, and the galactose/proton symporter of *E. coli*. **(B)** The schematic diagram of the complementation vectors expressing different sugar transporters.

In order to investigate the effects of different sugar transporters on the galactose metabolism of *S. thermophilus*, different sugar transporters were expressed in strain *S. thermophilus* Δ*lacS*, namely, the lactose permease of *S. thermophilus*, the N-terminal domain of the lactose permease of *S. thermophilus*, the lactose permease of *E. coli*, and the galactose/proton symporter of *E. coli*. The amino acids encoded by these proteins were 634 AA, 486 AA, 417 AA, and 464 AA, respectively (Figure 3A). Primers were designed to clone the above transporter-coding genes, fused to the constitutive promoter P32, and ligated into the expression vector pNZ8148. The structure diagram of the constructed recombinant expression vectors were shown in Figure 3B. These vectors were transformed into *E. coli*. The transformants of pNZ8148-*lacS*, pNZ8148-*N-lacS*, pNZ8148-*lacY*, and pNZ8148-*galR* were verified by colony PCR using forward primer *P32*-F and reverse primer *lacS*-R, *N-lacS*-R, *lacY*-R, and *galR*-R, respectively. The correct complement vectors were further electrotransformed into *S. thermophilus* Δ*lacS*. The recombinant strains *S. thermophilus* Δ*lacS*/ pNZ8148-*lacS*, *S. thermophilus* Δ*lacS*/ pNZ8148-*N-lacS*, and *S. thermophilus* Δ*lacS*/ pNZ8148-*galR* were obtained. However, no colony was formed in the plate containing *S. thermophilus* Δ*lacS* transformed by pNZ8148-*lacY*.

### 3.4 Complementation effect of the complete and N-terminal of *Streptococcus thermophilus* lactose permease

**Figure 4:**
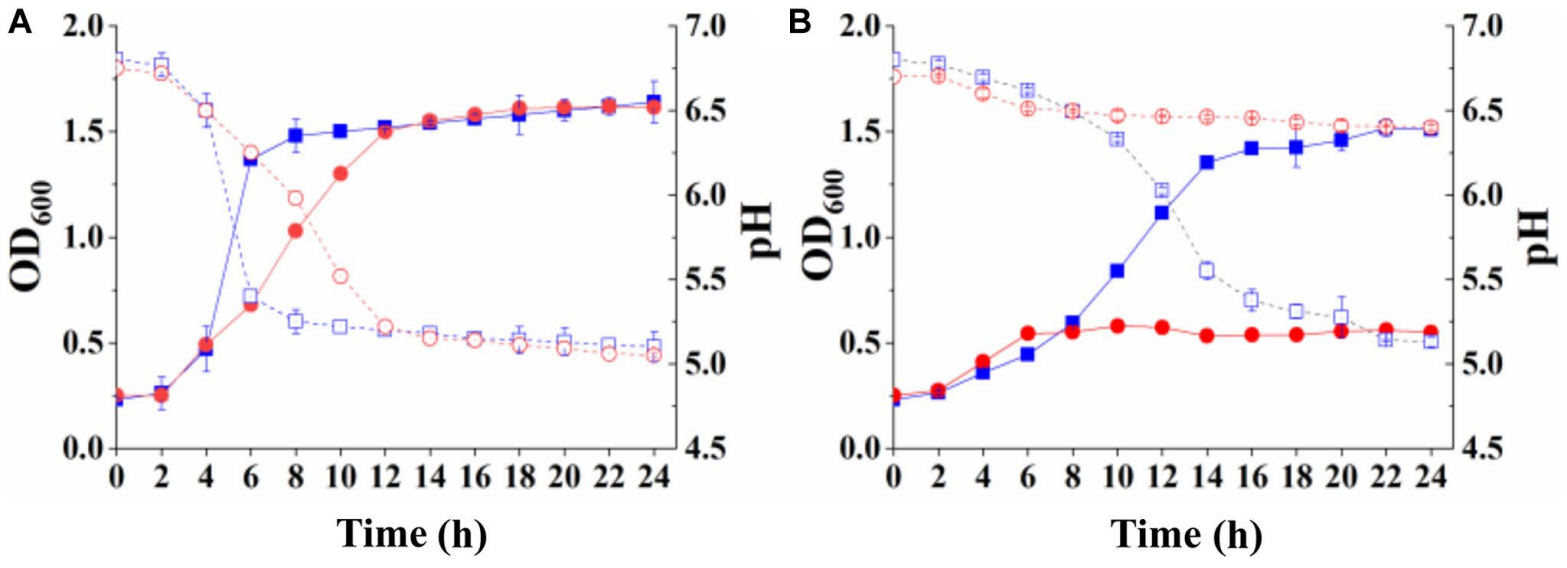
Growth curves (solid line) and pH changes (dot line) of recombinant *S. thermophilus* with complete lactose permease and its N-terminal. **(A)** *S. thermophilus* ΔlacS/pNZ8148-*lacS* cultured in LM17 (square) and M17-galactose medium (circle); **(B)** *S. thermophilus* ΔlacS/pNZ8148-*N-lacS* cultured in LM17 (square) and M17-galactose medium (circle).

The lactose and galactose utilization capacity of the recombinant strains was measured to determine whether the complete lactose permease or its N-terminal fragment from *S. thermophilus* could complement the deficiency in *S. thermophilus* Δ*lacS*. Strains *S. thermophilus* Δ*lacS*/pNZ8148-*lacS* and *S. thermophilus* Δ*lacS*/pNZ8148-*N-lacS* were cultured overnight in SM17 supplemented with 5 μg/mL chloramphenicol, then inoculated into LM17 and M17-galactose culture medium for 24 h. The growth results of the strains were shown in Figure 4. The recombinant strain *S. thermophilus* Δ*lacS*/pNZ8148-*lacS* recovered its growth ability in lactose and galactose medium, with OD_600_ values of 1.64 ± 0.10 and 1.62 ± 0.02, and pH values of 5.10 ± 0.09 and 5.05 ± 0.01, respectively (Figure 4A). *S. thermophilus* Δ*lacS*/pNZ8148-*N-lacS* recovered its growth ability in lactose medium, with OD_600_ value and pH reaching 1.51 ± 0.01 and 5.13 ± 0.03, respectively. However, this strain hardly grew in the galactose medium. The OD_600_ value and pH were 0.55 ± 0.01 and 6.40 ± 0.01, respectively (Figure 4B). These results indicated the full-length lactose permease could restore the lactose and galactose utilization capacity of strain *S. thermophilus* Δ*lacS*, while the N-terminal 1–486 amino acid fragment could only compensate for the lactose utilization capacity, but could not restore the galactose utilization capacity of the strain.

### 3.5 Complementation effect of the *Escherichia coli* galactose/proton symporter

**Figure 5:**
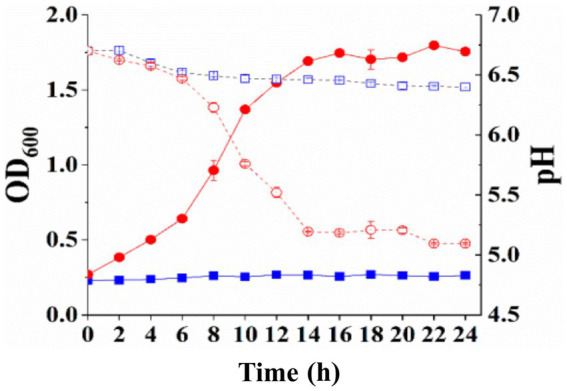
Growth curve (solid line) and pH change (dotted line) of recombinant *S. thermophilus* ΔlacS/pNZ8148-galR.

Since the transformation of *S. thermophilus* Δ*lacS* by the complement vector expressing *E. coli* lactose permease has not been successful, we only studied whether the galactose/proton symporter from *E. coli* could play a role in lactose permease-deficient mutant of *S. thermophilus*. The recombinant *S. thermophilus* Δ*lacS*/pNZ8148-*galR* was cultured overnight in SM17 supplemented with 5 μg/mL chloramphenicol, and then inoculated into LM17 and M17-galactose medium for 24 h, respectively. The growth status of the strain was shown in Figure 5. The recombinant strain expressing *E. coli* galactose/proton symporter recovered the growth ability in galactose medium, with OD_600_ of 1.76 ± 0.01 and pH value of 5.10 ± 0.01, respectively. However, the OD_600_ and pH values were 0.26 ± 0.01 and 6.40 ± 0.01 in the lactose medium, respectively. These results indicated that the galactose/proton symporter from *E. coli* could compensate for the galactose utilization deficiency of the lactose permease-deficient strain *S. thermophilus* Δ*lacS*, but not the lactose utilization.

## 4 Discussion

It has been reported that lactic acid bacteria ingest lactose or galactose in two ways. The first one is via lactose/galactose permease, in which lactose is hydrolyzed to glucose and galactose under the action of intracellular β-galactosidase. These two monosaccharides are then metabolized through the EMP and Leloir pathway, respectively (Devos and Vaughan, 1994). In addition, some lactic acid bacteria such as *Lactobacillus rhamnosus* (Tsai and Lin, 2006) and *Lactococcus lactis* (Neves et al., 2010) also have lactose-specific phosphotransferase systems and galactose-specific-phosphotransferase systems. This system phosphorylates lactose/galactose to lactose 6-phosphate/galactose 6-phosphate and transports it into the cell, where lactose 6-phosphate is hydrolyzed to glucose and galactose 6-phosphate by phospho-β-galactosidase. The two monosaccharides are further metabolized through EMP and tagatose-6-phosphate pathways, respectively (Devos and Vaughan, 1994). In *S. thermotropis*, lactose metabolic operon containing *lacS* and *lacZ* is obtained by horizontal gene transfer (Prajapati et al., 2013). In recent years, the function and mechanism of LacS for lactose transport in *S. thermophilus* have been elucidated. In this study, we constructed *S. thermophilus* Δ*lacS*, a lactose permease-deficient mutant of *S. thermophilus*. The strain lost the ability to use lactose. Subsequently, complement vectors expressing the complete lactose permease of *S. thermophilus* and its N-terminal 1–486 amino acid residues were constructed and transformed into *S. thermophilus* ΔlacS. By measuring the growth of the recombinant strains, it was found that the strains restored their growth ability in a lactose medium, which was consistent with previous studies that the C-terminal domain of LacS was not essential for lactose transport (Geertsma et al., 2005a,b).

According to the ability of *S. thermophilus* to metabolize galactose, it can be divided into two categories: galactose utilization type and galactose non-utilization type, of which the latter type accounts for the majority of reported *S. thermophilus* strains. Many strains of *S. thermophilus* used in the dairy industry also only metabolize the glucose portion of lactose and cannot utilize galactose, thus leading to the accumulation of galactose in fermented dairy products such as yogurt (Leong-Morgenthaler et al., 1991; Poolman, 2002). Residual galactose in dairy products will reduce the quality of fermentation products. For example, a high concentration of galactose will lead to cheese browning during heating, and residual galactose will also be used by allogenic fermentation agents to produce carbon dioxide, resulting in cheese cracking, fracture, and other tissue defects (Hutkins et al., 1986; Michel and Martley, 2001; Baskaran and Sivakumar, 2003). Excessive galactose in dairy products can also have adverse effects on human health, especially in patients with galactosemia, and excessive intake of galactose can lead to the accumulation of toxic galactitol in human tissues and cells (Novelli and Reichardt, 2000; Leslie, 2003). Exploring the reason why wild-type *S. thermophilus* uses galactose can provide a new strategy for screening and constructing strains with stable galactose fermentation ability. Previous studies have suggested that *S. thermophilus* does not utilize galactose due to the lack of enzymes for galactose metabolism in the cells. With the deepening of physiological and genetic research of *S. thermophilus* and the development of whole genome sequencing technology, the complete operon gene *galKTEM*, which encodes enzymes required for the Leloir pathway, can be predicted on all the *S. thermophilus* strains. However, galactose cannot be metabolized by most *S. thermophilus* strains (Vaughan et al., 2001; de Vin et al., 2005).

Several factors have been reported to be related to the galactose utilization capacity of *S. thermophilus*. The deficiency of galactose metabolism in *S. thermophilus* may be related to the base sequence of gal promoter. Vaughan et al. (2001) reported that galactose utilization phenotypic *S. thermophilus* mutated at three locations of the *galK* promoter, including base substitution at −9 and − 15 and single base insertion at −37. Anbukkarasi et al. (2014) found that galactose utilization phenotypic *S. thermophilus* had base substitution at the −9 position of the *galK* promoter, base substitution at the −25 position of the *galR* promoter, and base deletion at the −28 position. Although the gal promoter plays an important role, it is not the only factor determining the galactose phenotype of *S. thermophilus*. Hu et al. (2018) sequenced the *galR-galK* interval of 22 *S. thermophilus* strains and found that the galactose utilization phenotypes of *S. thermophilus* CS6, CS12, CS16, CS17, CS18, and CS19 had the same sequences as the galactose non-utilization phenotypes. The galactose phenotype of *S. thermophilus* may also be related to the level of protein translation. Despite the efficient transcription of the *gal* gene, most strains of *S. thermophilus* do not synthesize large amounts of galactokinase. Vaillancourt et al. (2002) showed that the similarity of *galKTE* gene between *S. salivarius* and *S. thermophilus* was as high as 95%, the *gal* promoter sequence of both was the same, and the transcription levels of *galT* and *galE* were similar. However, the galactokinase activity of *S. thermophilus* was much lower than that of *S. salivarius*. Further sequence analysis showed that there were two nucleotide differences between the ribosome binding site of *galK* of *S. thermophilus* and *S. salivarius*, resulting in a low translation level of the *galK* gene of *S. thermophilus* (Vaillancourt et al., 2004; de Vin et al., 2005). In this study, the strain expressing complete lactose permease in *S. thermophilus* Δ*lacS* recovered the growth ability in a galactose medium. However, the strain expressing the N-terminal of lactose permease recovered its growth ability only in lactose medium. This result confirmed that LacS in *S. thermophilus* not only played a role in transporting lactose, but also was responsible for galactose transport. Furthermore, it implied that the C-terminal domain of LacS was equally essential for galactose transport, which is inconsistent with lactose transport. By comparing the LacS protein sequence of different *S. thermophilus* strains in the NCBI database, amino acid mutations in the C-terminal domain were found, which may be one of the reasons for the galactose phenotypes of *S. thermophilus*.

*E. coli* is the most well-studied prokaryotic model organism to date. In the lactose operon of *E. coli*, structural genes of β-galactosidase, lactose permease, and galactoside transacylase are arranged on the genome in the order of *lacZ*, *lacY*, and *lacA, respectively* (Marbach and Bettenbrock, 2012). The lactose permease LacY transports lactose into the cell. The β-galactosidase LacZ breaks the galactoside bond of lactose to produce galactose and glucose. The galactoside transacylase LacA transfers the acetyl group from acetyl-CoA to β-galactoside. Mutations in either *lacZ* or *lacY* can produce a lactose non-utilization phenotype. The β-galactosidase in *S. thermophilus* has 1,026 amino acids, which is almost the same size as the β-galactosidase in *E. coli*. The sequence identity of these two proteins is 32%, and they both belong to the LacZ type β-galactosidase (Xu et al., 2022a,b). However, the molecular weight difference between the two lactose permeases LacS and LacY is large, and no homology is found by the sequence alignment. In this experiment, the transformation of *S. thermophilus* Δ*lacS* by the complement vector expressing lactose permease of *E. coli* has not been successful, which may be due to the toxicity caused by the membrane protein. *E. coli* can also efficiently metabolize galactose, and its metabolic pathway is also the Leior pathway. In addition, *E. coli* also has a specific transporter of galactose, namely galactose/proton symporter (Weickert and Adhya, 1993). No homolog of this protein was found in *S. thermophilus*. However, the complement vector with *E. coli* galactose/proton symporter could restore the galactose utilization of the strain *S. thermophilus* Δ*lacS*, but could not restore the lactose utilization.

The results of this study can be applied to the development of a food-grade expression vector of *S. thermophilus*. At present, there have been reports on the construction of food-grade expression vectors based on key genes of lactose operon in lactic acid bacteria chromosomes (Xu et al., 2022a,b; Wang et al., 2023). Maccormick et al. (1995) knocked out *lacF* gene from *Lactococcus lactis* MG5267 by double-exchange homologous recombination. The *lacF* gene is responsible for lactose transport, and *lacF*-deficient strains will produce a lactose non-utilization phenotype. The vector expressing LacF was complementary to the deficient strain, resulting in a lactose utilization phenotype. *S. thermophilus* is a potential cell factory for heterologous protein expression, but its food-grade expression vector is relatively rare. Sasaki et al. (2004) constructed a food-grade expression vector with the thymidylate synthase gene *thyA* as a selective marker for *S. thermophilus*. Spontaneous mutants of *thyA* of *S. thermophilus* were obtained in a medium supplemented with high concentrations of trimethylolpropane. However, this process requires the addition of thymidylate in the medium, which increases the cost of use. Labrie et al. (2005) demonstrated that α-galactosidase gene *aga* in *Lactococcus raffinolactis* ATCC 43920 can serve as an effective food-grade selective marker for *S. thermophilus*. However, α-galactosidase activity was inefficient at 42°C (the optimal growth temperature of *S. thermophilus*), and its expression was down-regulated in milk culture. Therefore, the marker can only be used to screen recombinant *S. thermophilus* strains in meliobiose medium at 37°C in the laboratory. In the future, sugar transporter for lactose/galactose can be used as a food-grade screening marker. The gene attached to a vector will complement *S. thermophilus* Δ*lacS*, leading to the restoration of lactose or galactose utilization and bacterial growth. On this basis, a new heterogenic expression tool of *S. thermophilus* will be developed using lactose or galactose as screening conditions. Given that lactose and galactose can be used as natural carbon sources by *S. thermophilus*, this food-gade expression system would not require additional substances to be added as screening pressure. In addition, if this system is applied to the fermentation of dairy products, the functional products such as antibodies and other proteins can be more easily delivered to the host.

## 5 Conclusion

A mutant *S. thermophilus* Δ*lacS* was constructed by deletion of the lactose permease coding gene of *S. thermophilus* A25, which is capable of utilizing lactose and galactose. *S. thermophilus* Δ*lacS* lost the ability to utilize lactose and galactose. Subsequently, different sugar transporters were expressed in this strain to detect their effects. The recombinant strain expressing complete lactose permease of *S. thermophilus* recovered the growth ability in lactose and galactose medium, while the strain expressing N-terminal of lactose permease recovered the growth ability only in lactose medium. The transformation of *S. thermophilus* Δ*lacS* was not successful with the complement vector expressing *E. coli* lactose permease, while the strain expressing *E. coli* galactose/proton symporter could recover its growth ability in galactose medium. These results suggested that the complete structure of the lactose permease in *S. thermophilus* played an important role in its galactose utilization, and that the galactose/proton symporter derived from *E. coli* also compensate for the galactose utilization of *S. thermophilus*. These functional transporters lay the foundation for future modification of galactose-utilizing *S. thermophilus* and for food-grade screening markers of expression vectors.

## Data availability statement

The original contributions presented in the study are included in the article/supplementary material, further inquiries can be directed to the corresponding authors.

## Author contributions

JZ: Formal analysis, Investigation, Methodology, Writing – original draft. YL: Formal analysis, Investigation, Software, Writing – original draft. SZ: Funding acquisition, Supervision, Writing – review & editing. ZX: Conceptualization, Funding acquisition, Supervision, Writing – review & editing.
